# Short-term safety, tolerability and efficacy of a very low-calorie-ketogenic diet interventional weight loss program versus hypocaloric diet in patients with type 2 diabetes mellitus

**DOI:** 10.1038/nutd.2016.36

**Published:** 2016-09-19

**Authors:** A Goday, D Bellido, I Sajoux, A B Crujeiras, B Burguera, P P García-Luna, A Oleaga, B Moreno, F F Casanueva

**Affiliations:** 1 Department of Endocrinology and Nutrition, Hospital del Mar, Barcelona, Spain; 2 Department of Medicine, Universitat Autonoma de Barcelona, Barcelona, Spain; 3 CIBER Fisiopatologia de la Obesidad y Nutricion (CIBERobn), Madrid, Spain; 4 Division of Endocrinology, Complejo Hospitalario Universitario de Ferrol and Coruña University, Ferrol, Spain; 5 Medical Department, Pronokal Group, Barcelona, Spain; 6 Division of Endocrinology, Department of Medicine, Complejo Hospitalario Universitario de Santiago (CHUS) and Santiago de Compostela University (USC), Santiago de Compostela, Spain; 7 CIBER Fisiopatologia de la Obesidad y Nutricion (CIBERobn), Madrid, Spain; 8 Endocrinology and Nutrition Department, Hospital Universitario Son Espases, Mallorca, Spain; 9 CAIBER-Investigation Unit, Hospital Universitario Son Espases, Mallorca, Spain; 10 Clinical Nutrition and Morbid Obesity Unit. Hospital Universitario Virgen del Rocio, Sevilla, Spain; 11 Endocrinology Department, Basurto Hospital, Bilbao, Spain; 12 Endocrinology and Nutrition Division, Hospital Universitario Gregorio Marañon, Madrid, Spain

## Abstract

**Brackground:**

The safety and tolerability of very low-calorie-ketogenic (VLCK) diets are a current concern in the treatment of obese type 2 diabetes mellitus (T2DM) patients.

**Objective:**

Evaluating the short-term safety and tolerability of a VLCK diet (<50 g of carbohydrate daily) in an interventional weight loss program including lifestyle and behavioral modification support (Diaprokal Method) in subjects with T2DM.

**Methods:**

Eighty-nine men and women, aged between 30 and 65 years, with T2DM and body mass index between 30 and 35 kg m^−^^2^ participated in this prospective, open-label, multi-centric randomized clinical trial with a duration of 4 months. Forty-five subjects were randomly assigned to the interventional weight loss (VLCK diet), and 44 to the standard low-calorie diet.

**Results:**

No significant differences in the laboratory safety parameters were found between the two study groups. Changes in the urine albumin-to-creatinine ratio in VLCK diet were not significant and were comparable to control group. Creatinine and blood urea nitrogen did not change significantly relative to baseline nor between groups. Weight loss and reduction in waist circumference in the VLCK diet group were significantly larger than in control subjects (both *P*<0.001). The decline in HbA1c and glycemic control was larger in the VLCK diet group (*P*<0.05). No serious adverse events were reported and mild AE in the VLCK diet group declined at last follow-up.

**Conclusions:**

The interventional weight loss program based on a VLCK diet is most effective in reducing body weight and improvement of glycemic control than a standard hypocaloric diet with safety and good tolerance for T2DM patients.

## Introduction

Medical nutritional therapy aiming at weight loss is a mainstay of treatment for obese subjects with type 2 diabetes mellitus (T2DM).^1^ An interplay between human obesity and T2DM was strongly confirmed in numerous epidemiological studies^2^ and both diseases are rapidly growing in parallel worldwide with major health consequences. In fact, weight loss has been associated with an improvement not only in glycemic control but also in other cardiovascular risk factors commonly altered in subjects with T2DM.^3, 4^ Nonetheless, long-term non-pharmacologic weight loss interventions for adults with T2DM have shown limited efficacy.^5^ Thus, alternative weight loss strategies that are safe and effective in subjects with T2DM are in need.

The optimal degree of caloric restriction and macronutrient distribution of medical nutritional therapy in T2DM is not well defined. A systematic review of weight loss interventions in subjects with T2DM revealed that interventions including very low-calorie diets (VLCD) along with moderate physical activity and behavioral intervention produced the largest effect.^5^ Although the number of randomized clinical trials assessing the efficacy of VLCD in subjects with T2DM is limited, data suggest considerable weight loss, improved beta-cell function, and improved quality of life associated with short-term VLCD.^5, 6, 7, 8, 9, 10, 11^ However, in 2008 the American Diabetes Association stated as part of its nutrition recommendations for diabetes that VLCD appeared to have limited utility in the treatment of T2DM and should only be considered in conjunction with a structured weight loss program.^12^ On the other hand, evidence suggests that there are not an ideal percentage of calories from carbohydrate, protein and fat for all people with diabetes; therefore, macronutrient distribution should be based on individualized assessment of current eating patterns, preferences and metabolic goals. Although numerous studies have attempted to identify the optimal mix of macronutrients for the meal plans of people with diabetes, recent systematic review^13^ found that there is no ideal mix that applies broadly for successful weight loss in subjects with T2DM and that macronutrient proportions should be individualized.^14^ It has been claimed that high-protein diets may help promote weight loss, maintain lean body mass, and improve lipid and plasma glucose profiles in obese subjects with our without T2DM^15, 16, 17, 18^ and prevent hepatic steatosis in obese animal models.^19^ However, concern has been raised that increased protein intake, could cause deterioration of renal function particularly in those with microalbuminuria or established diabetic nephropathy,^20^ and that high-protein interventions are not feasible in a 'real-world setting.^21^ In addition, short-term studies have shown that reducing total carbohydrate intake is associated with improved insulin sensitivity and glycemic control.^13^ Conversely, current standards of care of the American Diabetes Association for the subject with T2DM state that the recommended daily allowance for digestible carbohydrate is 130 g per day to provide adequate glucose as the required fuel for the central nervous system without reliance on glucose production from ingested protein or fat.^1^

Against this background, the primary aim of our study was to evaluate the short-term safety and tolerability of a low-carbohydrate, ketogenic diet (<50 g of carbohydrate daily; VLCK diet) as part of an interventional weight loss program including lifestyle and behavioral modification support (Diaprokal Method) in subjects with T2DM. As secondary aims, we compared weight loss and changes in metabolic parameters between subjects following the interventional weight loss program or a low-fat hypocaloric diet together with a lifestyle and behavioral modification program made available by the health-care provider.

## Subjects and methods

### Subjects

Eighty-nine men and women participated in our prospective, open-label, multi-centric randomized clinical trial with a duration of 4 months and parallel group design. Eligibility criteria for the study included age between 30 and 65 years, previous diagnosis of T2DM and body mass index between 30 and 35 kg m^−^^2^. Exclusion criteria included duration of T2DM longer than 10 years, insulin therapy, hemoglobin A1c (HbA1c) ⩾9% and fasting C-peptide <1 ng ml^−1^. In addition, subjects presenting with impaired renal function (defined as an estimated glomerular filtration rate <60 ml min^–1^ per 1.73 m^2^), impaired liver function (defined as liver enzymes greater than equal to twofold the upper normal limit), alcohol intake ⩾40 g per day for men and ⩾24 g per day for women, pregnancy, lactation, or severe eating or psychiatric disorder according to the investigator criterion were excluded from the study.

Study participants were recruited in the Endocrinology departments of seven participating Centers across Spain. Centralized approval was granted by the Ethics Committee of one of the participating Centers (Institut Municipal d'Assistència Sanitària, Hospital del Mar) and thereafter ratified by the local Ethics Committee at each participating site. Written informed consent was obtained from all study participants prior to randomization. Randomization to one of the two study groups was stratified by participating Center.

### Study design and dietary interventions

The 4-month dietary intervention in subjects randomly assigned to the interventional weight loss following a VLCK diet (VLCK diet group) as part of a commercial weight-loss program (DiaproKal Method) based on a high-biological-value protein preparations diet and natural foods or to a low-calorie diet (LC diet group) based on the ADA (American Diabetes Association) guidelines.^1^

The intervention for both groups included an evaluation by the specialist physician conducting the study, an assessment by an expert dietician, group meetings and exercise recommendations. Individual counseling to support lifestyle and behavioral modification throughout the study was performed according to a structured support program by an endocrinologist and a registered dietitian at each participating center in the LC diet group. The registered dietitian in the VLCK diet group was an employee of the company supporting the interventional program and used the same structured support plan as in the LC diet group. The program included nine individual sessions and a telephone contact every 15 days in both study arms.

### VLCK diet

The methodology in VLCK diet group was similar to that used in another recently published study evaluated the efficacy of a VLCK diet as part of a commercial weight loss program (Pronokal Method) in obesity.^22^ Each protein preparation contained 15 g protein, 4 g carbohydrates, 3 g fat and three specific active ingredients, (20 μg chromium, 0.8 g Ginseng and 0.4 mg Biotin); and provided 90–100 kcal. This method has three stages: active, metabolic stabilization and maintenance. The active stage consists of a very low-calorie diet (600–800 kcal per day), low in carbohydrates (<50 g daily from vegetables) and lipids (only 10 g of olive oil per day). The amount of high-biological-value proteins ranged between 0.8 and 1.2 g per each Kg of ideal body weight, to ensure meeting the minimal body requirements and to prevent the loss of lean mass. This method produces three ketogenic phases. In phase 1, the patients eat high-biological-value protein preparations five times a day, and vegetables with low glycemic index. In phase 2, one of the protein servings is substituted by a natural protein (for example, meat and fish) either at lunch or at dinner. In the phase 3, a second serve of the natural protein low in fat substituted the second serve of biological protein preparation. Throughout these ketogenic phases, supplements of vitamins and minerals, such as K, Na, Mg, Ca and omega-3 fatty acids, were provided in accordance to international recommendations. This active stage is maintained until the patient loses most of weight loss target, ideally 90%. Hence, the ketogenic phases were variable in time depending on the individual and the weight loss target, but they lasted between 30 and 45 days in total.

In the metabolic stabilization stage, the ketogenic phases were ended by the physician in charge of the patient based on the amount of weight lost, and started a low-calorie diet. At this point, the patients underwent a progressive incorporation of different food groups and participated in a program of alimentary re-education to guarantee the long-term maintenance of the weight lost. The maintenance stage consists of an eating plan balanced in carbohydrates, protein and fat. Based on each individual's basal metabolic rate as determined by the Harris Benedic equation, the calories consumed ranged between 1500 and 2250 kcal per day and the target was to maintain the lost weight and promote healthy life styles.

### LC diet

The LC diet was aimed at a daily energy restriction of 500–1000 kcal according to each individual's basal metabolic rate. Macronutrient dietary composition aimed at a daily intake of <30% of calories coming from fat, 10–20% from protein and 45–60% from carbohydrates.

### Safety and tolerability assessment

Safety parameters included renal function (plasma creatinine, blood urea nitrogen, urinary albumin-to-creatinine ratio and estimated Glomerular Filtration Rate using the Modification of Diet in Renal Disease study equation MDRD-eGFR), liver function (alanine aminotransferase, aspartate aminotransferase and total bilirubin) and plasma uric acid, sodium and potassium. These parameters were performed using automatic standard procedures (Cobas c711, Roche-Spain) and a Coulter LH 750 Hematology Analyzer, (Beckman Coulter, Inc.; Brea CA, USA). Beta-hydroxibutirate was measured from capillary blood (Optium Xceed Blood Glucose and Ketone Monitoring System; Abbott Laboratories, Chicago, IL, USA). The method performed to detect microalbuminuria was the albumin/creatinine ratio (μg mg^−1^) measured in spot urine samples. Diagnosis of microalbuminuria was defined when the spot collection was 30–300 μg mg^−1^ creatinine.

Safety parameters were assessed at baseline and at 2 weeks, 2 months (visit 5) and 4 months (visit 9, end of the study) following randomization. Capillary ketones were assessed at each study visit. Tolerability was assessed as the percentage of patients completing the 6–10 weeks pre-defined period of VLCK diet, and the incidence of pre-defined or unexpected adverse events (AE) throughout the study period.

### Anthropometrical and biochemical assessment

Body weight, body mass index and waist circumference were performed according to previously describe standardized procedures.^22^

As glucose homeostasis parameters fasting plasma glucose, HbA1c and insulin were quantified. The HOMA-IR (Homeostasis Model Assessment for Insulin Resistance) was estimated as previously reported^23^ and a HOMA-IR>3.2 was considered as indicative of insulin resistance.^22^ Lipid profile analysis included fasting plasma triglycerides and total-, low-density lipoprotein and low-density lipoprotein cholesterol.

Dietary adherence and patient satisfaction were assessed by the Eating Self-Efficacy Scale and Liker Scale (1=very unsatisfied, 2=unsatisfied, 3=indifferent, 4=satisfied, 5=very satisfied), respectively. Changes in the laboratory parameters were performed using automatic standard procedures (Cobas c711, Roche-Spain, Madrid, Spain) and a Coulter LH 750 Hematology Analyzer, (Beckman Coulter, Inc.) and were calculated as the difference between the baseline values and those at the end of the study.

### Statistical analysis

Sample size was calculated based on a previously reported 7% occurrence of AE in subjects participating in a randomized clinical trial evaluating weight-loss dietary interventions differing in macronutrient composition.^24^ Accordingly, a sample size of 38 subjects per group was estimated necessary to validate the hypothesis that the occurrence of AE would be equivalent in the two study groups, with an alpha error of 0.05 and a statistical power of 80%. A dropout rate of 15% was anticipated in both study groups. Thus, we aimed at recruiting a total of 45 subjects per group.

Statistical analysis was performed using Statistical Analysis System software (version 9.2; SAS Institute Inc., Cary, NC, USA). Analysis of the safety and tolerability (safety population) variables was performed with an intention-to-treat analysis with baseline or last observation carried forward when the complete set of data for an individual was not available. Changes in body weight, BMI and waist circumference between groups were compared in the 'efficacy population', composed by those with at least one efficacy measurement available after randomization. Data on continuous variables are expressed as mean±s.d. unless stated otherwise. Categorical variables are described as percentage and number of valid observations. Other secondary measures were compared between groups at each study visit. No imputations for missing values were performed. Differences between groups were evaluated using parametric or non-parametric test as appropriate (*χ*^2^ or Fisher's test for categorical variables, and analysis of variance or Mann–Whitney *U*-test). Statistical significance was set at a *P*-value <0.05.

## Results

### Baseline characteristics of patients

The main clinical characteristics of the study participants are shown in Table 1. A total of 89 subjects were randomized to the low-calorie, ketogenic diet (VLCK diet) group (*n*=45) or the usual care low-calorie (LC diet) group (*n*=44). Attrition by completion of study visits was not different between groups (VLCK diet: 11.1% (5/45), LC diet: 18.2% (8/44); *P*=0.384). Anthropometric and metabolic parameters at baseline were comparable between the two study groups (Table 1).

### Diet-induced changes in safety parameters

**Figure 1:**
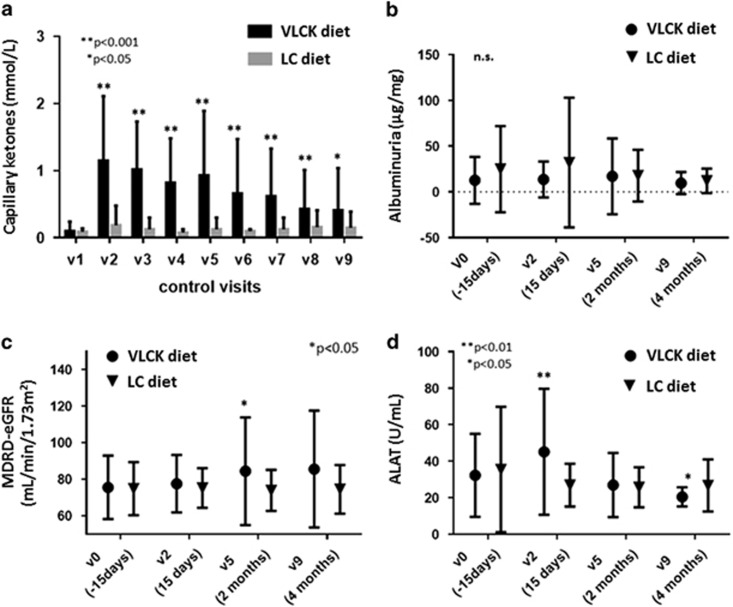
Diet-induced changes in safety parameters in the very low-calorie-ketogenic (VLCK) diet and low-calorie (LC) diet groups. (**a**) Changes in capillary ketones. (**b**) Changes in albuminuria. (**c**) Changes in estimated Glomerular Filtration Rate using MDRD study equation (MDRD-eGFR). (**d**) Changes in ALAT. *P-value<0.05: all cases, between-group comparisons conducted by ANOVA.

As expected by design, capillary blood β-hidroxibutirate concentration was larger in the VLCK diet group over the VLCK diet time period and for the remaining of follow-up (Figure 1a). Ketonemia positive (⩾0.3 mmol l^−1^) were detected in 91.1% of subjects of VLCK diet group during follow-up. The largest mean capillary ketonemia in the VLCK diet group during the study was recorded at 2 weeks follow-up (1.15±0.96 mmol l^−1^). The study participant with a ketonemia of 4.2 mmol l^−1^ did not present a random glucose >250 mg dl^−1^ or a pH<7.3. Despite this fact, no significant differences in the laboratory safety parameters were found between the two study groups (Figure 1). Changes from baseline in the urinary albumin-to-creatinine ratio (Figure 1b) and estimated Glomerular Filtration Rate using the Modification of Diet in Renal Disease study equation (Figure 1c) in the VLCK diet group were not statistically significant through the intervention period, and no differences were observed between the two weight loss strategies. Regarding to microalbuminuria diagnosis (UARC⩾30–300 μg mg^−1^), it was present in 6.3% in the VLCK diet group and in 17.6% of the LC diet group without reach statistically significant differences between groups (*P*=0.156) at the end of the study. Likewise, creatinine and blood urea nitrogen did not change significantly within study groups at the 2- or 4 months evaluations relative to baseline nor between groups (data not shown). Alanine aminotransferase and aspartate aminotransferase were slightly albeit significantly larger in the VLCK diet group as compared with the LC group at 2 weeks (alanine aminotransferase: 45.16 vs 26.85 IU ml^−1^, *P*<0.005; aspartate aminotransferase: 38.53 vs 22.15 IU ml^−1^, *P*<0.001) but not at the end of follow-up (4 months), (Figure 1d). Percentage of subjects in the VLCK diet group who presented with alanine aminotransferase or aspartate aminotransferase plasma concentration threefold higher than the upper limit of the normal range was not significantly different compared with controls (0% *P*=0.157). Bilirubin plasma concentration remained invariable all over the study and did not differ between groups. At all-time points, sodium, potassium, chloride, calcium and magnesium remained stable and within the normal limits in the two study groups. Finally, mean uric acid level was larger in the VLCK diet group at 2 weeks (*P*=0.021), but not at 2- or 4 months (data not shown).

Among the 45 subjects allocated to the VLCK diet group, 7 (15.6%) discontinued the low-carbohydrate, ketogenic diet (<50 g of carbohydrate daily) before 6 weeks whereas 29 (64.4%) completed at least the pre-defined maximum of 10 weeks. No serious AE were reported. Mild AE were reported by 80% of the VLCK diet subjects as compared with 41% of the subjects in the control group (Table 2; *P*<0.001). Among the pre-defined AE, asthenia, headache, nausea and vomiting were more common in VLCK diet group at 2 weeks (all *P*<0.05). The number of subjects reporting these AE in the VLCK diet group declined at last follow-up. At the end of the study, constipation (*P*<0.005) and orthostatic hypotension (*P*<0.05) were more commonly referred by subjects in the VLCK diet group (respectively, *n*=8 and *n*=6) compared with control subjects (both, *n*=0). Not pre-defined AE were more frequent in the VLCK diet group at 2 weeks but not at 4 months (Table 2). Only one patient in the VLCK diet group discontinued the study because of an AE consisting of nausea associated with ketosis, a patient for not obesity related surgery and the rest by personal choice.

### Diet-induced changes in efficacy parameters

At 4 months, weight loss and reduction in waist circumference in subjects in the VLCK diet group were significantly larger than in control subjects (both *P*<0.001; Table 3). At completion of the study, >85% of the VLCK diet subjects achieved a weight loss >10% relative to baseline. Fasting plasma glucose decreased significantly in the two study groups (both *P*<0.05 relative to baseline), although the decline in HbA1c was statistically significant only in the VLCK diet group (*P*<0.0001; Table 3). Relevantly, insulin sensitivity as assessed from HOMA-IR at the end of follow-up was statistically lower than in LC diet group (3.51 vs 4.61; *P*<0.05). Regarding to plasma lipid profile at 4 months, no statistically significant changes were observed in total cholesterol, LDL-C and HDL-C in both diet groups, but the VLCK diet induced a statistically significant decrease in triglycerides (*P*=0.004), which was not observed in the LC diet group (Table 3).

Dietary adherence as assessed from the Eating Self-Efficacy Scale was comparable between the two study groups. Finally, patients in the VLCK diet group rated more satisfactory the weight loss intervention they had been allocated to. At 4 months, 92.5% of the participants in the VLCK diet group and 68.5% in the control group deemed the intervention satisfactory or very satisfactory (*P*=0.005).

## Discussion

Our data show that VLCK diet (a low-calorie-ketogenic diet, <50 g of carbohydrate daily) as part of a interventional weight loss program including lifestyle and behavioral modification support over a 4-month period is a safe, well tolerated, and accepted medical nutritional therapy option for subjects with T2DM. Furthermore, VLCK diet intervention in subjects with T2DM is associated with significantly larger weight loss along with amelioration of glycemic control as compared with a standard care nutritional intervention based on the ADA guidelines. The short-term efficacy of an intense caloric restriction as that reported herein for weight loss in T2DM and before bariatric surgery is well established.^5, 6, 7, 8, 9, 10, 11^ Our study adds to the field on the potential validity of increasing the protein content and decreasing the carbohydrate content in a VLCK diet as a safe and effective approach to medical nutritional therapy in T2DM.

The optimal mix of macronutrients of medical nutritional therapy for people with T2DM remains unsolved.^1, 13^ However, although consensus is lacking, diets high in protein are commonly seen as less appropriate for subjects with T2DM specially if micro- or macro-albuminuria are present because of the concept that reducing protein intake appears to slightly slow progression to renal failure.^13, 25^ Our data show that a 30–53% daily caloric content as protein does not result in increased appearance or worsening of albuminuria, nor deterioration of plasma creatinine over the course of a 4-month intervention, neither changes in eGFR in T2DM subjects with or without albuminuria but without chronic kidney disease at baseline. These findings are similar to previous report that evidenced that a low-carbohydrate diet is as safe as Mediterranean or low-fat diets in preserving renal function among moderately obese participants with or without T2DM.^26^

It has been proposed that diets aiming at weight loss that are high in protein may be advantageous because of increased satiety despite negative energy balance, and sustained basal energy expenditure despite body weight loss due to a sparing of fat-free mass.^27^ Thus, the relatively high-percent protein content of our dietary plan could be viewed as protein sparing. That is, a strategy to avoid the ensuing reduction of total daily protein intake associated with energy restricted diets.^27^ Admittedly, the percent daily protein intake in our study subjects corresponds to 1.0–1.6 g of protein intake/actual body weight/day. Thus, it is of note, that lack of detrimental effect on renal parameters in our series was found in the context of larger protein intakes than those tested in clinical trials examining the effects of varying amounts of daily protein intake in subjects with or without diabetic kidney disease at baseline.^13, 28, 29^

The macronutrient mix used in the VLCK diet group is also characterized by carbohydrate content well below the 130 g recommended daily allowance,^1^ throughout the 6–10 initial weeks (32–89 g carbohydrate per day). VLCK diets have been shown to have beneficial effects on weight loss, insulin sensitivity and HbA1c in most studies.^13, 30^ A study in which 84 patients with obesity and T2DM were randomized to either a low-carbohydrate, ketogenic diet or a low-glycemic, reduced-calorie diet over a 24-week period in patients with obesity and T2DM, showed diet lower in carbohydrate led to greater improvements in glycemic control (hemoglobin A1c, fasting glucose, fasting insulin) and weight loss, and more frequent medication reduction/ elimination than the low glycemic index diet.^31^

A low-carbohydrate intake results in a lower circulating insulin/glucagon ratio, which promotes a high level of serum non-esterified fatty acids used for oxidation and resulting in production of ketone bodies. Accordingly, periodic testing of capillary ketones yielded higher values in subjects in the VLCK diet group as compared with those in the LC diet group, with 91.1% of subjects with positive ketonemia (only the values of β-hidroxibutirate ⩾0.3 mmol l^−1^). However, in all but one of the subjects in the VLCK diet group capillary beta-hydroxibutirate concentration remained lower than that typically observed in diabetic ketoacidosis in type 1 diabetic subjects.^32^ The reasons for such a markedly elevated ketonemia in this study participant remain elusive. Biochemical data ruled out diabetic ketoacidosis (glycemia remained below the range of acute decompensation), and intercurrent illness, excessive alcohol intake and intense exercise were also excluded.

Achievements of our medical nutritional therapy intervention included a significant higher weight loss and improvement in metabolic control. The weight loss effectiveness of our approach is supported by the findings of 98 and 85% of our study subjects achieving a >5% or >10% weight loss at the end of follow-up. Of note, the 15% weight loss relative to baseline in subjects allocated to the VLCK diet group in our study is larger than that reported in the intensive lifestyle intervention arm of the Look Ahead trial.^33^ Furthermore, our medical nutritional therapy strategy resulted in marked improvement of glycemic control. Our study design does not allow disentangling of the relative effects of weight loss or restricted carbohydrate intake.^13^ However, it is worth emphasizing that the likelihood of achieving HbA1c<7% was twofold in those allocated to the VLCK diet group. This increased reduction of HbA1c in the intervention group, could be explained by an improvement in the insulin sensitivity as demonstrated by the improvement in the HOMA-IR at the end of the study. In fact, the VLCK diet induced a decrease in triglycerides, in line with the improvement in glycemic control as plasma levels of triglycerides is a biomarker of dysfunctional insulin sensitivity.^30, 34^

Importantly, the metabolic beneficial effects occurred in the absence of serious AEs. Moreover, the observed AEs associated with VLCK diet were in line of those previously associated with very-low carbohydrate interventions.^35^ Of note, only one patient in the VLCK diet group discontinued the study because of an AE with ketosis and 15.6% of the subjects in the VLCK diet group presented early termination of the low-carbohydrate-ketogenic diet period. Attrition rate in our study was similar to that previously reported in VLCK diet, high-protein or very-low carbohydrate diets.^23, 36, 37^ Moreover, the proportion of subjects that deemed the intervention satisfactory was higher in subjects in the VLCK diet group.

The short duration of our study is a limitation. However, the main goal of the current study was to evaluate safety and tolerability in subjects with T2DM of the phases in our method with the largest energy- and carbohydrate-restriction along with the higher proportion of calories as protein.

In summary, our study demonstrates the short-term feasibility, safety, tolerability and efficacy of an interventional weight loss program (Diaprokal Method) as medical nutritional therapy in subjects with T2DM. This medical nutritional therapy intervention resulted in significant weight loss in most study participants, along with marked amelioration of glycemic control as compared with a standard of care nutritional intervention based on the ADA guidelines. The long-term safety and efficacy of the proposed medical nutritional therapy strategy warrants further evaluation.

## Figures and Tables

**Table 1:** Baseline characteristics of patients

	*All patients*	*VLCK diet group*	*LC diet group*	P*-value*^a^
	N*=89*	N*=45*	N*=44*	
Men/women (*N*)	31/58	15/30	16/28	0.7642
Age (years)	54.53 (8.37)	54.89 (8.81)	54.17 (7.97)	0.6876
Weight (kg)	90.51 (11.37)	91.47 (11.43)	89.54 (11.37)	0.4255
Height (cm)	165.15 (8.98)	165.52 (8.62)	164.76 (9.43)	0.6928
BMI (kg m^−2^)	33.07 (1.56)	33.25±1.52	32.88 (1.60)	0.2611
Waist circumference (cm)^b^	107.04 (8.54)	108.13 (8.55)	105.94 (8.49)	0.2317
Fasting blood glucose (mg dl^−1^)	139.77 (39.43)	136.87 (34.43)	142.81 (44.26)	0.4825
HbA1c (%)	6.89 (1.06)	6.89 (1.11)	6.88 (1.03)	0.9743
HbA1c⩾7%^c^	36 (40.9%)	21 (46.7%)	15 (34.9%)	0.2611
HOMA index^b^	6.36 (3.78)	6.87 (4.39)	5.78 (2.90)	0.1933
Insulin resistance (HOMA index >3.2)c,^b^	71 (85.5%)	39 (88.6%)	32 (82.1%)	0.3945
Creatinine (mg dl^−1^)	0.91 (0.23)	0.90 (0.17)	0.92 (0.28)	0.7412
Urea (mg dl^−1^)	36.68 (10.42)	35.93 (8.62)	37.48 (12.12)	0.4933
MCRD-eGFR (ml min^−1^ per 1.73 m^2^)	75.24 (15.97)	75.58 (17.41)	74.89 (14.50)	0.8411
UACR (μg mg^−1^)	18.51 (37.67)	12.71 (25.57)	24.88 (47.09)	0.1355
Microalbuminuria (UACR ⩾30–300 μg mg^−1^)^c^	12 (14.0%)	4 (8.9%)	8 (19.5%)	0.1556
Uric acid (mg dl^−1^)	5.23 (1.32)	5.26 (1.29)	5.20 (1.36)	0.8336
Uric acid>7.0 mg dl^−1^	7 (8.0%)	3 (6.7%)	4 (9.5%)	0.8353
ALAT (U ml^−1^)	33.85 (28.90)	32.27 (22.75)	35.47 (34.28)	0.6094
ASAT (U ml^−1^)	25.95 (12.06)	28.00 (14.07)	23.86 (9.30)	0.1100
*Therapy for T2DM*^c^				0.1260
Oral antidiabetic^c^	71 (79.8%)	33 (73.3%)	38 (86.4%)	
Lifestyle modification^c^	18 (20.2%)	12 (26.7%)	6 (13.6%)	

Abbreviations:

ALAT, alanine aminotransferase; ASAT, aspartate aminotransferase; BMI, body mass index; HOMA, Homeostasis Model Assessment for Insulin Resistance; LC diet, low-calorie diet; MCRD-eGFR, estimated glomerular filtrate rate by Modification of Diet in Renal Disease equation; T2DM, type 2 diabetes mellitus; UACR, urinary albumin/creatinine ratio; VLCK diet, very low-calorie-ketogenic diet.

a ANOVA or *χ*^2^-tests according to the type of data.

b Not measured in all patients: 88 waist circumference, 83 HOMA index and insulin resistance.

c Number and percentage. All other values are mean (s.d.).

**Table 2:** Adverse effects in both groups

*Symptoms*^a^	*V2 (15 days)*			*V9 (4 months)*		
	*VLCK diet group (*n*=45)*	*LC diet group (*n*=44)*	P*-value*	*VLCK diet group (*n*=45)*	*LC diet group (*n*=44)*	P*-value*
Asthenia	7	0	0.0092	1	0	0.3396
Headache	9	1	0.0124	2	0	0.1739
Nausea	9	0	0.0028	3	0	0.0936
Vomiting	7	0	0.0092	1	0	0.3396
Constipation	2	0	0.1772	8	0	0.0046
Cramps	1	0	0.3429	0	0	–
Myalgia	1	0	0.3429	1	0	0.3396
Muscular weakness	1	1	0.9328	0	0	–
Heaviness and tiredness of legs	1	1	0.9328	0	0	–
Hair loss	1	0	0.3429	2	0	0.1739
Orthostatic hypotension	0	0	–	6	0	0.0155
Edema	0	0	–	1	0	0.3396
Othersb,^c^	20	4	0.0004	5	8	0.0731
						
*Patients lost*						
Due to side-effects	0	0		1	0	
Voluntary dropout	0	4		4	4	
Total dropout	–	–		5	8	

Abbreviations: LC diet, low-calorie diet; VLCK diet, very low-calorie-ketogenic diet

.

a All values show the number of patients.

b Other adverse effects described in V2: anxiety, cold, diarrhea, epigastric pain, shoulder pain, halitosis, hunger, hypoglycemia, sachets intolerance, bad taste, dizziness, bloating, paresthesia, dry mouth, allergic rhinitis, nasal trauma.

c Other effects described in V9: cold, abdominal pain, back pain, halitosis, diarrhea, urinary tract infection, increased blood pressure, palpitations.

**Table 3:** Efficacy outcomes

	*VLCK diet group (*n*=45)*			*LC diet group (*n*=40)*		
	*Baseline*	*4 months*	P*-value*^a^	*Baseline*	*4 months*	P*-value*^a^
*Body weight*						
Body weight (kg)	91.5 (11.4)	76.8 (9.1)	**<0.0001**	90.0 (11.3)	84.95 (13.6)	0.5960
Weight lost>5% of weight	–	40 (97.6%)	–	–	18 (50.0%)^**†**^	–
Weight lost>10% of weight	–	35 (85.4%)	–	–	6 (16.7%)^**†**^	–
BMI (kg m^−2^)	33.3 (1.5)	27.9 (1.8)	**<0.0001**	32.9 (1.6)	31.0 (2.2)	**<0.0001**
Waist (cm)	108.1 (8.6)	96.1 (7.6)	**<0.0001**	105.8 (8.5)	100.4 (9.2)	**0.0481**
						
*Glycemic control*						
Fasting glycemia (mg dl^−1^)	136.9 (34.4)	108.9 (20.4)	**<0.0001**	140.5 (43.1)	123.3 (24.3)	0.1821
HbA1c (%)	6.9 (1.1)	6.0 (0.7)	**<0.0001**	6.8 (1.0)	6.4 (0.8)	0.1453
Patients with HbA1c ⩾7%	21 (46.7%)	5 (12.8%)	**0.0008**	15 (34.9%)	9 (25.7%)	0.3828
HOMA Index	6.9 (4.4)	3.5 (1.9)	**<0.0001**	5.8 (2.9)	4.6 (2.5)^**†**^	**0.0010**
Patients treated with oral antidiabetic drugs	33 (73.3%)	20 (50.0%)	**0.0267**	38 (86.4%)	30 (83.3%)	0.7057
						
*Lipid profile*						
Total cholesterol (mg dl^−1^)	200.1 (36.0)	187.5 (46.3)	0.1615	199.4 (51.0)	191.7 (34.1)	0.4489
Triglycerides (mg dl^−1^)	150.5 (54.4)	114.6 (57.2)	**0.0040**	176.1 (92.0)	158.3 (61.0)	0.3308
LDL-c (mg dl^−1^)	112.7 (33.6)	110.6 (38.4)	0.7892	109.8 (45.5)	107.1 (29.9)	0.7629
HDL-c (mg dl^−1^)	55.9 (11.1)	54.5 (11.3)	0.5728	55.1 (11.7)	52.4 (10.0)	0.3017

Abbreviations: HDL, high-density lipoprotein; HOMA, Homeostasis Model Assessment for Insulin Resistance; LC diet, low-calorie diet; LDL, low-density lipoprotein; VLCK diet, Very low-calorie-ketogenic diet.

Changes in weight and metabolic control at 4 months (V9)

a Statistically significant differences from baseline and ^†^between groups (*P*<0.05) assessed by ANOVA or *χ*^2^-tests according to the type of data.

Bold values indicate statistically significant data (*P*<0.05).
